# Select Whole-Cell Biofilm-Based Immunogens Protect against a Virulent Staphylococcus Isolate in a Stringent Implant Model of Infection

**DOI:** 10.3390/vaccines10060833

**Published:** 2022-05-24

**Authors:** Stephen J. Dollery, Janette M. Harro, Taralyn J. Wiggins, Brendan P. Wille, Peter C. Kim, John K. Tobin, Ruth V. Bushnell, Naomi J. P. E. R. Tasker, David A. MacLeod, Gregory J. Tobin

**Affiliations:** 1 Biological Mimetics, Inc., Frederick, MD 21702, USA; wiggins@bmi-md.com (T.J.W.); john.tobin@bmi-md.com (J.K.T.); bushnell@bmi-md.com (R.V.B.); tasker@bmi-md.com (N.J.P.E.R.T.); dmacleod8@gmail.com (D.A.M.); tobin@bmi-md.com (G.J.T.); 2 Department of Microbial Pathogenesis, School of Dentistry, University of Maryland, Baltimore, MD 21201, USA; jharro@umaryland.edu (J.M.H.); brendan.wille@umaryland.edu (B.P.W.); pkim@som.umaryland.edu (P.C.K.)

**Keywords:** MRSA, staphylococcus, biofilm, vaccine, prosthetic implant, clearance, protection

## Abstract

Many microbes of concern to human health remain without vaccines. We have developed a whole-microbe inactivation technology that enables us to rapidly inactivate large quantities of a pathogen while retaining epitopes that were destroyed by previous inactivation methods. The method that we call UVC-MDP inactivation can be used to make whole-cell vaccines with increased potency. We and others are exploring the possibility of using improved irradiation-inactivation technologies to develop whole-cell vaccines for numerous antibiotic-resistant microbes. Here, we apply UVC-MDP to produce candidate MRSA vaccines which we test in a stringent tibia implant model of infection challenged with a virulent MSRA strain. We report high levels of clearance in the model and observe a pattern of protection that correlates with the immunogen protein profile used for vaccination.

## 1. Introduction

*Staphylococcus aureus* is a gram-positive bacterium associated with a range of serious acute and chronic diseases, including bacteremia, skin and soft tissue infections, pneumonia, endocarditis, urinary tract infections, osteomyelitis, and surgical and medical implanted device infections [1,2,3]. *S. aureus* resistance to methicillin was first reported in 1961 [4,5] and methicillin-resistant *S. aureus* (MRSA) quickly became a leading cause of healthcare-acquired (HA) infections [6]. Due to the rapid proliferation of multiple antibiotic resistance markers by this microbial species and its propensity to change from acute to chronic and recurrent infections, *S. aureus* remains a critical concern in any setting where colonization of the host is possible. In recent years, HA-MRSA infections have declined due to heightened countermeasures, but community-acquired MRSA cases have remained stable [7,8]. The CDC reports that MRSA caused >320,000 infections in hospitalized patients and >10,000 deaths during 2017 in the United States [9], and other estimates are even higher (>19,000 deaths [7]).

Pathogenically, *S. aureus* mediates a wide range of disease by differentially expressing a vast array of virulence factors that initiate colonization and growth, drive tissue damage, and promote immune evasion [2]. Chronic disease is partly the result of biofilm growth which develops when bacteria adhere to either host tissue or an abiotic surface and encapsulate themselves in a protective, extracellular polymer matrix that is largely impenetrable to antibiotics [2,10,11]. Biofilm-mediated infections are up to 500 times more resistant to the immune system and to treatment with antibiotics than planktonic bacteria [11]. Biofilm-mediated infections often require surgical debridement and prolonged aggressive antibiotic therapy. As therapeutic options for treating *S. aureus* are increasingly limited, new antibacterial interventions are needed. Prophylactic vaccines against *S. aureus* would have enormous impact in the healthcare fight against antibiotic-resistant strains.

The development of anti-*S. aureus* vaccines and interventions has been frustrated by several characteristics of *S. aureus,* including functional redundancy of virulence factors, differential expression of proteins, heterogeneity in biofilm, and lack of genetic conservation amongst strains [12,13].

Vaccine development against *S. aureus* has moved to a multivalent approach to compensate for the numerous issues highlighted above. Multivalent vaccines containing multiple subunit proteins have shown improvements in efficacy [14,15,16]. However, strain variation in proteins (i.e., SdrD and SdrE) reduces the protective efficacy of the vaccines [17]. Although clinical trials with polyvalent compositions are in progress, the large number of potential antigenic targets complicate the selection of subunits for a prophylactic vaccine. The failure of subunit vaccines has shifted the focus to vaccine approaches using multiple antigens/virulence factors, whole bacteria, or whole-cell lysates [18]. 

The sterilization of pathogens with gamma and UVC irradiation are attractive approaches for the development of inactivated whole-organism vaccines [19]. However, irradiation typically destroys immunogenic epitopes needed to stimulate protective immune responses. A minor fraction of the damage results from gamma and UVC radiation depositing energy that directly damages macromolecules, while the vast majority of damage results from indirect damage by reactive oxygen species (ROS) formed by the radiolysis of water or an unidentified source from within the bacteria [20,21,22,23,24]. To overcome epitope damage, we have developed a method: irradiation in the presence of the powerful antioxidant manganese-decapeptide-phosphate (MDP), derived from the extreme radioresistant bacterium Deinococcus radiodurans. When bacteria are mixed with MDP and exposed to supralethal doses of γ-rays or UVC irradiation, their genomes are destroyed, but antigenic epitopes remain intact [25,26]. In the presence of MDP, the epitopes are protected and can still stimulate immune protection. The method produces highly immunogenic preparations [25,27]. The first-generation gamma-irradiated (Ir)-MRSA vaccine (community associated-MRSA based) stimulated protective immunity to subcutaneous MRSA challenge in a mouse model, significantly decreasing the abscess size and bacterial burden compared to mice immunized with either phosphate-buffered saline (PBS) or MRSA irradiated without MDP [25].

Major hurdles for MRSA vaccine development include variably expressed antigen targets between phases of growth (e.g., biofilm versus planktonic), the large number of potential combinations of antigens in a multimeric subunit vaccine, and antigenic variation of potentially protective subunits. In response to these problems, we have developed a system for testing the immunogenicity of multiple preparations of whole-cell bacteria that express the most protective immunogens from specific phases of growth. We combined the preparation of these immunogens with the most recent advances in irradiation-inactivation technology to enhance the potency of the immunogens and thereby vaccinate mice with epitopes that may never have been presented previously in a vaccine. Using a stringent implant model of biofilm infection, we induced unusually high levels of clearance in mice challenged with the virulent MRSA M2 strain. The patterns of protection between immunogen groups are reproducible and provide rationale for the further development of vaccines.

## 2. Materials and Methods

### 2.1. Growth of Bacterial Cultures

**Figure 1:**
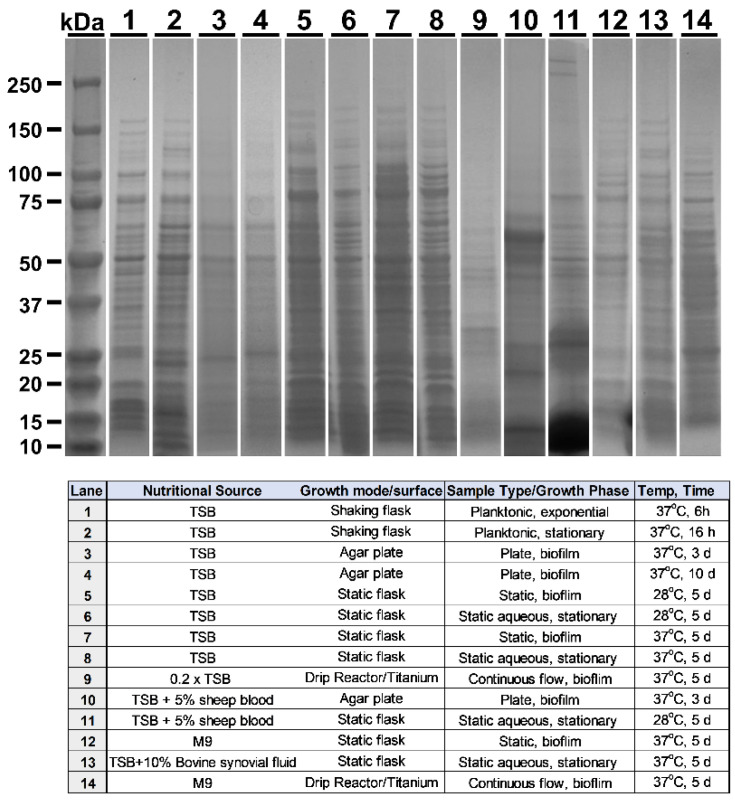
MRSA growth conditions yield unique protein profiles. **Top**: Coomassie-stained SDS-PAGE of lysates. **Bottom**: key to numbered lanes providing details of growth parameters.

Isolate MRSA-M2 (M2) of methicillin-resistant *Staphylococcus aureus* was isolated from an osteomyelitis patient undergoing treatment at the University of Texas Medical Branch (Galveston, TX, USA) [28]. M2 cultures were propagated using multiple methods with the intent of differentially expressing a variety of antigenic proteins among the various cultures. MRSA-M2 was cultured as follows to yield unique protein profiles, and the culture numbers correspond to the lane numbers in Figure 1 (e.g., in lane 1, culture 1 was run). All cultures were propagated using atmospheric gas. In the case of culture 1 (planktonic), 500 µL of overnight starter culture was subcultured into 100 mL of Tryptic Soy Broth (TSB, BD Bacto, Becton Dickenson, Sparks, MD, USA) and grown at 37 °C. The cultures were agitated at 180 rpm in an orbital shaker and harvested at 6 h during exponential growth. Culture 2 (planktonic): growth conditions were the same as those of culture 1; however, harvest was at 16 h, during the stationary phase. Culture 3 (plate biofilm): cells were grown at 37 °C on Trypticase Soy Agar (TSA) (BBL TSA II Becton Dickenson, Sparks, MD, USA) for 3 days. Culture 4 (plate biofilm): cells were grown at 37 °C as a biofilm on thick TSA plates for 10 days. Culture 5 (static aqueous biofilm): cells were cultured in motionless T182 tissue culture flasks (Celltreat, Pepperell, MA, USA) while submerged under 50 mL of TSB at 28 °C for 5 days. TSB was replaced at day 3. Adherent cells were harvested. Culture 6 (static aqueous suspension): cells were grown in a static motionless suspension as in culture 5. Non-adherent cells from the suspension were harvested at day 5 (2 days post media replacement). Cultures 7 and 8 were cultured in motionless flasks as in cultures 5 and 6; however, the temperature was increased to 37 °C. Culture 9 (Titanium plate drip reactor biofilm): cells were grown via continuous flow drip reactor (Biosurface Technologies Corporation, Bozeman, MT, USA). For batch phase, 10 mL of 1 × 10^7^ colony-forming units (CFUs) per mL were inoculated into drip reactor chambers and cultured overnight with no angle in a 37 °C incubator. For continuous flow phase, reactors were inclined to an angle of 10 degrees and chambers were supplied with 2 g/L of TSB (1/15th) and 2 g/L of D-glucose at a flow rate of 240 µL per minute for 5 days (days 2–6 of culture). Culture 10 (plate biofilm): cells were cultured as in 3; however, TSA was supplemented with 5% sheep’s blood (Thermo Scientific Blood Agar, Thermo Fisher Scientific, Frederick, MD, USA). Culture 11 (static aqueous suspension): cells were grown as in culture 6, and supplemented with 5% sheep’s blood. Non-adherent cells from the suspension were harvested. Culture 12 (static aqueous biofilm): cells were cultured with M9 media as in culture 7. Culture 13 (static aggregate suspension): cells were grown as in culture 6; however, TSB was supplemented with 10% bovine synovial fluid (Articular Engineering, Northbrook, IL, USA). Where possible, the removal of aggregate clusters was avoided during media replenishment at day 3. Non-adherent cells from the suspension were harvested. Culture 14 (Titanium plate drip reactor biofilm): cells were grown in a drip reactor as in culture 9; however, M9 media (BD Difco, Becton Dickenson, Sparks, MD, USA) was used for nourishment. All cultures were collected directly (suspension) or were scraped into cold PBS (Gibco, Gaithersburg, MD, USA) with a cell scraper and resuspended. Cultures were pelleted for 15 min at 2000× *g* at 4 °C and washed twice in PBS before proceeding. For titration of CFU, cells were serially diluted in TSB and plated on TSA.

### 2.2. Protein Analysis of Bacterial Cultures

Samples of bacteria grown in varying conditions were normalized for the number of cells and the protein profiles were analyzed using denaturing polyacrylamide gels (SDS-PAGE). Briefly, 50 µL samples containing approximately 1 × 10^6^ bacterial cells were mixed with an equal volume of 2× Laemmli SDS-PAGE reducing sample buffer and heated for 20 min at 85 °C. The samples were vortexed vigorously and 10 μL samples were electrophoresed in 8–16% polyacrylamide gradient gels (Biorad, Hercules, CA, USA). After electrophoresis, the gels were either stained for total protein visualization using Coomassie Brilliant Blue R-250 or electro-transferred to nitrocellulose membranes for immunoblotting. After transfer, immunoblots were blocked with a solution of 10% non-fat dried milk in PBS, pH 7.5 supplemented with 0.2% Tween-20 (PBS-T), probed with mouse anti-MRSA antiserum (as indicated in the figure legends) diluted in PBS-T with 5% milk, washed in PBS-T, and detected with an anti-mouse-HRP secondary antibody conjugate (Seracare, Gaithersburg, MD, USA), washed again in PBS-T, and visualized using enhanced chemiluminescent reagent (Pierce Biotechnology, Rockford, IL, USA) and X-ray film (BIOMAX Light Film; Kodak, Rochester, NY, USA).

### 2.3. Carbonylation Assay

Protein oxidation of irradiated bacteria was examined using the OxyBlot Protein Oxidation Detection Kit (S7150) (Chemicon International, Thermo Fisher Scientific, Frederick, MD, USA). Bacterial samples that were UVC-treated with or without the MDP complex were denatured and derivatized to 2,4-dinitrophenylhydrazone (DNP-hydrazone) by reacting with 2,4-dinitrophenylhydrazine (DNPH) as per the manufacturer’s protocol. Samples were electrophoresed in 8–16% gradient polyacrylamide gels as described above. The proteins were electro-transferred to nitrocellulose. The membranes were incubated with primary rabbit antibody, specific to the DNP moiety of the proteins (Chemicon International, Thermo Fisher Scientific, Frederick, MD, USA). After washing, the membranes were probed with HRP-conjugated secondary goat anti-rabbit IgG as directed. Proteins were visualized with chemiluminescent reagent (Pierce Biotechnology, Rockford, IL, USA) and imaged by exposure to light-sensitive films (BIOMAX Light Film; Kodak, Rochester, NY, USA).

### 2.4. Murine Prosthetic Implant Infection Model

Inbred C57BL/6 mice (6 to 8 weeks old) were purchased from Jackson Laboratories (Bar Harbor, ME, USA). The mice were maintained under microisolator conditions in the animal facility at the University of Maryland School of Medicine (Baltimore, MD, USA), in accordance with protocols reviewed and approved by the Institutional Animal Care and Use Committee (IACUC). The mice were vaccinated by intramuscular injection at weeks 0 and 3 with either vehicle alone or UVC-MDP-inactivated bacterial preparations (2.5 × 10^7^ CFUs) with Alum as the adjuvant. On week 6, the mice were anesthetized via i.p. injection of 100 mg ketamine/kg of body weight) and 10 mg xylazine/kg (Rugby Laboratories, Inc., Rockville Center, NY, USA). The left leg of each mouse was cleansed and a sterile 0.25-mm insect pin (Fine Science Tools, Foster City, CA, USA) was surgically implanted through the tibia, according to the methods previously described by Li et al. and Prabhakara et al. [29]. In this study, 1 µL of inoculum was pipetted onto the exposed ends of the pin, which corresponds to an ID90 in this model. On week 7, the mice were euthanized and the tibiae were harvested and homogenized. Tissue homogenates were serially diluted and plated on *S. aureus* selective media, CHROMagar (CHROMagar, Paris, France). Bacterial burdens were enumerated from the plates and calculated as CFUs/mg bone with a limit of detection of 100 CFUs. Studies were performed with a methicillin-resistant *S. aureus* (MRSA) clinical isolate M2 obtained from the University of Texas Medical Branch (Galveston, TX, USA). The strain was grown on Trypticase Soy Agar (TSA) with 5% sheep blood (ADD) supplemented with 0.3 mg/mL oxacillin and Tryptic Soy Broth (TSB) (ADD). The bacterial inoculum was prepared from mid-logarithmic cultures grown for 3 h at 37 °C following a 1:100 subculture of an overnight MRSA-M2 culture into fresh TSB. The bacteria were washed with PBS and the target inoculum of 3000–5000 CFUs per 1 µL was prepared by adjusting the bacterial suspension based on optical density and known concentration values.

### 2.5. UVC-Inactivation of Bacterial Replication Capability

Solutions of bacteria at 1 × 10^9^ CFU per mL were prepared for irradiation with the addition of 1 mM MnCl2, 3 mM DP1 (synthetic decapeptide (DP1) H-Asp-Glu-His-Gly-Thr-Ala-Val-Met-Leu-Lys-OH), and 25 mM potassium phosphate buffer, pH 7.4 (MDP) to form a protective MDP complex. 0.2 mL volumes of MDP-bacteria were placed in thin-wall 0.5 mL tubes normally used for polymerase chain reactions (PCR). The tubes were capped and placed onto a UVC light source emitting 4.5 mW/cm^2^ for 90 s. Prior to use in immunization studies, the UVC-treated bacteria were tested rigorously for retention of residual replication activity by plating samples derived from at least 1 × 10^9^ CFU on agar plates. The plates were incubated at 37 °C overnight and examined for the presence of bacterial colonies.

### 2.6. Statistical Analyses

Pearson’s Chi-squared and Kruskal–Wallis rank sum tests were calculated. All statistical analyses were performed using R version 3.6.2. (http://www.r-project.org/, accessed on 24 March 2022) with the exception of standard error calculations. Standard error and graphing were performed using GraphPad Prism version 8.0.0. (San Diego, CA, USA).

## 3. Results

### 3.1. Evaluation of Proteomic Differences between Culture Conditions

M2 MRSA was grown under various conditions for the expression and evaluation of phase-specific proteomes. The culture conditions were selected based on their potential to provide unique protein profiles, their similarity to in vivo infection (e.g., blood or synovial fluid), and the use of diverse culture platforms (e.g., shaker/planktonic vs. drip reactor/biofilm). Fourteen of the culture conditions used are summarized in the bottom of Figure 1 (bottom), the resultant protein profiles of which were visualized via Coomassie stain, and the representative images of which are shown in Figure 1.

**Figure 2:**
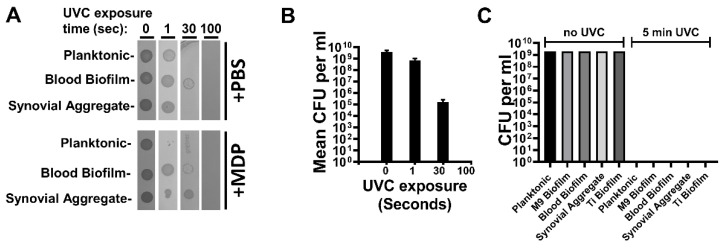
UVC irradiation kills MRSA. (**A**) MDP has minimal impact on MRSA survival following UVC exposure: 100 µL vials of MRSA were exposed to UVC for the indicated times at 2 × 10^8^ per tube and spotted onto LB-agar plates to observe residual colony formation activity. Each spot correlates with 2 × 10^6^ CFU of bacteria prior to UVC treatment. (**B**) Quantitation of data shown in Panel A. Means with SEM are shown. (**C**) Bar graph showing CFU per mL from samples before and after 5 min of UVC exposure with MDP. Data are representative of >5 independent UVC exposure experiments (depending on the sample). 4 × 10^8^ CFU equivalents were plated to check viability.

**Figure 3:**
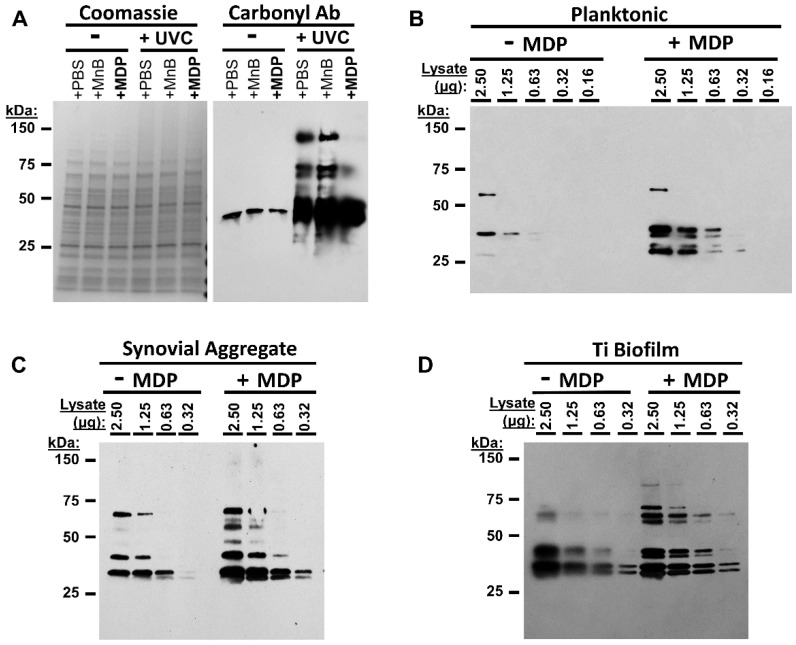
MDP protects MRSA proteins during UVC irradiation. (**A**) MDP protects proteins from oxidation. Planktonic MRSA were prepared in PBS, Mn+ buffer, or with MDP and subjected to 5 min UVC exposure or not. Lysates were prepared and analyzed via either (left) Coomassie stain (concentration control) or (right) western blot for derivatized carbonyl groups (DNP). (**B**–**D**) Planktonic, synovial fluid or titanium drip culture preparations of M2 were irradiated for 5 min with MDP or buffer, lysed, and remaining epitopes were analyzed via western with anti-MRSA mouse sera raised against inactivated whole-cell planktonic MRSA.

**Figure 4:**
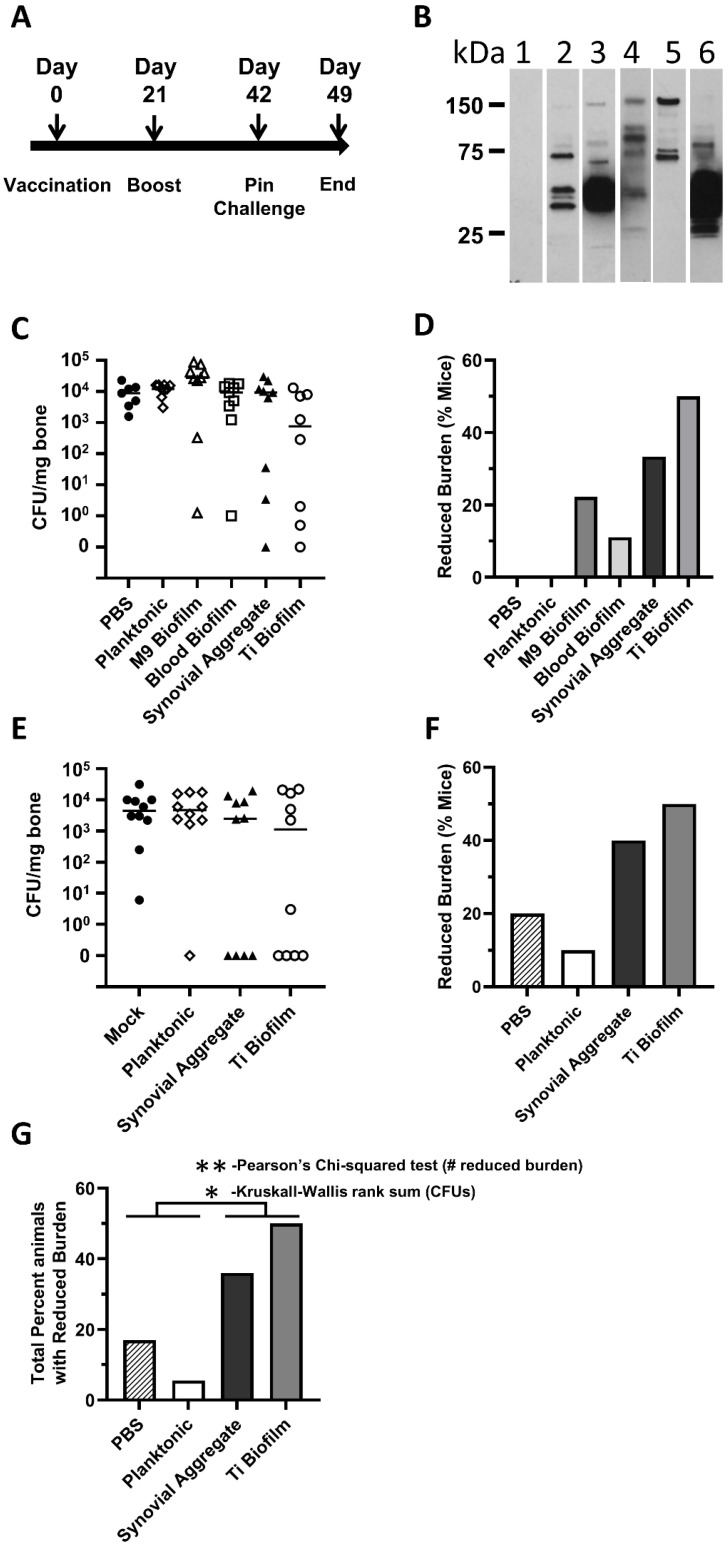
Infected-bone-implant model. (**A**) Mice were vaccinated on day 0 and boosted on day 21. Mice were challenged on day 42 and observed for 7 days post-challenge. CFU in the tibia following implant was determined per mg of bone. (**B**) Western blot of MRSA (planktonic) probed with sera from mouse groups (pre-challenge/post-boost). Lanes 1 and 2 were probed with sera from mice immunized with 16hr planktonic cultures. In Lanes 3–6, sera were probed with sera from single mice that were later shown to be protected in the following order; 3, M9 Biofilm; 4, Blood Biofilm; 5, Synovial Aggregate; 6, Titanium Biofilm. (**C**) Study 1 (9 mice per group); scatter plot of bacterial burden as CFUs per mg of bone from mice vaccinated with different whole-cell preparations and challenged. (**D**) Study 1; percent mice with a reduced burden of bacteria to lower than 10^4^ CFU per mg bone. (**E**) Study 2 (10 mice per group); scatter plot of bacterial burden (CFUs per mg of bone) from mice vaccinated with different whole-cell preparations and then challenged. (**F**) Study 2; percent mice with a reduced burden of bacteria to lower than 10^4^ CFU per mg bone. (**G**) Combined analysis Protection was significantly elevated for the Synovial Aggregate and Ti Biofilm (** indicates a Pearson’s Chi-squared test *p*-value of 0.002. * Indicates a Kruskal–Wallis rank sum test *p*-value of 0.03). Note: in A three mice were omitted prior to challenge, two from the PBS group, one from the Ti Biofilm group).

For further analysis as potential immunogens for vaccination, five conditions were selected based upon their unique expression profiles in combination with directed selection (Lanes 2, 9, 11, 12, 13: termed Planktonic, Ti Biofilm, Blood Biofilm, M9 Biofilm, and Synovial aggregate (respectively) in Figure 2, Figure 3 and Figure 4. Directed selection criterion included the following: (1) biofilms grown on titanium (Ti) may mirror post-surgical implant infection and contain critical protective epitopes. (2) MRSA is known to thrive in protein-rich environments and has hemolytic genes, so biofilms were grown in media including sheep’s blood as these cultures may contain critical epitopes. (3) MRSA is also known to form dramatic bio-aggregates when grown in synovial fluid [30,31], so aggregate cultures grown in synovial fluid were selected as a possible source of unique epitopes that may make protective immunogens. (4) In contrast, MRSA cultures grown as a biofilm under minimal nutrient conditions are known to adapt to growth in stringent conditions, and was so selected for it’s potential to provide unique epitopes. (5) Finally, a standard planktonic culture grown in nutrient-rich conditions was selected as a further diverse condition with a unique protein profile. These cultures represent a diverse set of growth conditions, and each condition yielded a unique protein profile.

### 3.2. Presence of MDP during UVC Irradiation: Effects on Bacterial Survival, Protein Oxidation, and Protection of Epitopes

Bacterial growth capability is readily extinguished by exposure to UVC irradiation. It has been previously shown that MRSA inactivated by exposure to gamma-radiation in the presence of a complex of manganese, decapeptide, and phosphate (MDP) results in preparations with a greater number of native epitopes than bacterial irradiated without MDP [25]. We sought to: (A) establish UVC inactivation conditions for MRSA, (B) observe the effect of the presence of MDP on survival of MRSA during UVC irradiation, and (C) determine whether epitopes were retained to a greater extent in the presence of the MDP complex. Selected MRSA preparations were irradiated with a UVC lamp (4.5 mW/cm^2^) in the presence or absence of the MDP complex (Figure 2A,B). In each instance, the CFU per mL of MRSA-M2 was over 1 × 10^9^ initially and declined to zero after a 100 s exposure, indicating rapid and complete inactivation. For each of the preparations, the inactivation kinetics was equivalent in the presence or absence of MDP, indicating that MDP did not enhance survival, consistent with previous observations that MDP does not protect against direct nucleotide damage [25,26,32]. A five-minute UVC exposure was selected for subsequent in vitro and in vivo experiments to give a large safety margin. Figure 2C shows CFU counts from preparations selected for use in vaccination experiments both before and after neutralization in the presence of MDP.

To determine whether MDP protected epitopes in the selected cultures during UVC exposure, we performed Coomassie staining and carbonylation testing (Figure 3A) and epitope analysis via western blot (Figure 3B–D). Although UVC irradiation without MDP did not appreciably alter the overall protein profiles (Figure 3A left), oxidative damage, as detected by carbonyl analysis, increased when the bacteria were irradiated without MDP (Figure 3A right). Carbonyl groups were more readily detected following exposure to UVC without MDP, consistent with oxidative damage occurring during UVC exposure. The presence of MDP during irradiation protected the sample from the same level of damage seen in the other irradiated samples (top bands). To examine UVC-induced epitope damage, detection with anti-MRSA antibodies was performed (Figure 3B,C). In each instance, several additional bands were detected in samples that had been irradiated in the presence of MDP, and bands were visible at lower concentrations. This indicates that under the selected conditions, differences in the immunogenic properties of the preparations were readily observable and that MDP+ preparations retained greater immunoreactivity.

### 3.3. Efficacy of Irradiated Whole-Cell S. aureus Vaccines in a Prosthetic Implant Model of Infection

The five selected preparations were tested for protection in a murine bone–implant challenge model. The UVC-MDP-inactivated bacterial preparations and PBS control were emulsified in Alum to elicit a more robust Th2 response and administered to mice using the immunization and challenge schedule as described in Figure 4A. As a simple way to visualize differences in antibody production between groups, sera from mice vaccinated with the preparations was used to probe gels prepared with lysates of planktonic cultures (Figure 4B). In each case, the sera recognized distinct bands indicating different immune responses following vaccination. In a first study, the bacterial burden in the infected tibiae were enumerated at one-week post-challenge. Mice with >10^4^ CFU/mg of bone were considered to have a reduced burden as this fell outside of the range of CFUs seen in the mock vaccinated group and in mice where vaccination appeared to have little effect; the average reduction of CFUs in mice that appeared to respond to vaccination was 3 logs (Figure 4C,D). The reduction was least potent/absent with the planktonic vaccines, while the greatest reduction was seen in animals vaccinated with biofilm and bioaggregate cultures. The reduction of CFU in the synovial and Ti-plate immunogen groups was the greatest of all. These results are consistent with the idea that immunogens harboring similar protein profiles to those encountered during challenge induce the best protection. We believe the titanium-grown biofilm is antigenically most similar to the epitopes present during this infection model. To test the reproducibility of protection, the study was replicated with select immunogens (Figure 4E,F). In this study, an even greater level of reduction in CFU was observed, with complete clearance of bacteria seen in 40–50% of the mice for the immunogen prepared from bacteria grown on titanium (one mouse with 3 CFU was included as cleared). In Figure 4E,F the general pattern of protection was almost identical to the first study (Figure 4D,E). A statistical analysis of the data reveals that the distribution of the data was irregular and unequal between groups, violating, for example, multiple assumptions of ANOVA, such as normality and homoscedasticity. We therefore applied nonparametric methods of analysis. Wilcoxson Rank sum pairwise comparison yielded significant protection in Synovial (*p* = 0.039) and Ti Drip groups (*p* = 0.039) versus PBS. However, because of the number of conditions tested, the protection observed did not quite meet test significance when correcting for multiple comparisons (*p* = 0.059). For these reasons we performed a Kruskal–Wallis rank-sum test (on CFU values) which determines if the samples originate within the same distribution. With this, we see a significant difference of *p* = 0.03 (Figure 4G). In addition, a Pearson’s Chi-squared test of numbers of mice with reduced burdens gives a *p*-value of 0.002 for a comparison of Titanium Biofilm and synovial aggregate to PSB with planktonic. We believe these data and analyses indicate that the titanium biofilm and the synovial aggregate vaccine candidates significantly reduce the burden of bacteria in mice.

## 4. Discussion

Biofilms of many pathogens including Staphylococcus are recalcitrant to antibiotic treatment and clearance in the host. In this study, we observed high clearance of infection in immunized mice using a stringent implant model of MRSA challenge. Whole-cell preparations of bacteria, propagated to yield divergent protein profiles, were inactivated in a process that ablates replicative function but retains a high level of protective immunogens. We and others have been harnessing this approach to generate whole-cell vaccines that have increased immunogenicity with promising results [25,26,32]. In our application of the UVC-inactivation method with poliovirus, we have observed up to 1000 times more epitope units per mg of immunogen when MDP is included during irradiation [32]. In an antibacterial vaccine, an increase in specific epitope presentation may allow for a reduced dose, minimizing the unwanted effects mediated by pathogen-associated molecular patterns (PAMPs) or damage-associated molecular patterns (DAMPs), and pattern-recognition receptors (PRRs) including Toll-like receptors (TLR) etc. 

Vaccines that target single bacterial immunogens have proven to be suboptimal for several reasons. Functional redundancy of targets has stymied vaccination efforts, with notorious examples including the multiple iron acquisition systems [12,33,34,35,36]. Immune targeting of one protein or toxin may allow a redundant alternative to function in disease. Differential expression of proteins during the multiple phases of growth could render the elicited immune responses useless during a second phase of growth [16]. Additionally, virulence factors may be ineffective vaccine targets if they are not conserved amongst all strains [17]. As a result, monovalent subunit vaccines designed against several *S. aureus* proteins have shown incomplete protection in animals, despite being highly immunogenic [37,38,39,40,41,42] and eliciting antibodies with effective opsonophagocytosis activity [43]. In contrast, a whole-cell vaccine presents a large number of immune targets, many of which contain genetically conserved epitopes.

As an alternative to protein targets, vaccine strategies have been tested against *S. aureus* polysaccharide immunogens (e.g., polysaccharide capsules, exopolysaccharide, and peptidoglycan), but again fail to protect [44,45,46,47,48]. Other vaccine strategies to target biofilm phenotypes have focused on the matrix encapsulating the bacteria, specifically the staphylococcal polysaccharide intercellular adhesin (PIA) composed of polysaccharide poly-N-acetyl-a-1,6-glucosamine (PNAG). Again, PNAG vaccine studies showed only partial protection, possibly due to PNAG shedding [46,49,50,51]. 

To address many of these issues, the present approach uses a novel whole-cell inactivation method that retains native epitopes that stimulate protective immunity. 

Consistent with previous findings and our hypotheses, immunogens that mimicked the challenge model afforded greater protection (reduced CFU burden and clearance of infection) than those which did not; the immunogens also provided a rich array of potential epitopes for recognition. A greater number of mice might have cleared infection if the challenge model incorporated an inoculation regime that mimicked biofilms at an earlier stage of polymer exo-matrix formation or that better mirrored the low number of CFU that may initiate biofilm patches. Both hypotheses can be tested in later experiments. 

Analysis of the differences in composition between forms of immunogens that do and do not protect can be used to identify correlates of immunity. The inclusion of varying planktonic and biofilm growth conditions, which mimic specific phases of natural infection, in inactivated whole-cell immunogens appears critical as the starting point for identifying immunogenic-subunit correlates of protective immunity. In future studies, we plan to perform these types of analyses to identify potential subunit candidates that can be combined into multimeric vaccine candidates.

We believe that the UVC-inactivated whole-cell vaccine platform is an extremely promising approach for generating immunogens that were previously technically challenging. In this study, we have demonstrated promising levels of protection and opened numerous avenues for the development of novel vaccines. 

## 5. Patents

A provisional patent has been submitted.

## Data Availability

Not applicable.
